# Lifespan Modulation in Mice and the Confounding Effects of Genetic Background

**DOI:** 10.1016/j.jgg.2014.06.002

**Published:** 2014-09-20

**Authors:** Lorna Mulvey, Amy Sinclair, Colin Selman

**Affiliations:** Institute of Biodiversity, Animal Health and Comparative Medicine, College of Medicine, Veterinary and Life Sciences, Graham Kerr Building, University of Glasgow, Glasgow G12 8QQ, UK

**Keywords:** Ageing, Dietary restriction, Genotype, Longevity, AL, *Ad libitum*, DR, dietary restriction, IIS, insulin/insulin like growth factor-1 signalling, RI, recombinant inbred, TOR, target of rapamycin

## Abstract

We are currently in the midst of a revolution in ageing research, with several dietary, genetic and pharmacological interventions now known to modulate ageing in model organisms. Excitingly, these interventions also appear to have beneficial effects on late-life health. For example, dietary restriction (DR) has been shown to slow the incidence of age-associated cardiovascular disease, metabolic disease, cancer and brain ageing in non-human primates and has been shown to improve a range of health indices in humans. While the idea that DR's ability to extend lifespan is often thought of as being universal, studies in a range of organisms, including yeast, mice and monkeys, suggest that this may not actually be the case. The precise reasons underlying these differential effects of DR on lifespan are currently unclear, but genetic background may be an important factor in how an individual responds to DR. Similarly, recent findings also suggest that the responsiveness of mice to specific genetic or pharmacological interventions that modulate ageing may again be influenced by genetic background. Consequently, while there is a clear driver to develop interventions to improve late-life health and vitality, understanding precisely how these act in response to particular genotypes is critical if we are to translate these findings to humans. We will consider of the role of genetic background in the efficacy of various lifespan interventions and discuss potential routes of utilising genetic heterogeneity to further understand how particular interventions modulate lifespan and healthspan.

## Introduction

The ageing process is characterised by progressive loss in cellular homeostasis and a decline in physiological function (Balcombe and Sinclair, 2001), resulting in an overall decline in fecundity and increased risk of mortality over time. While ageing was historically believed to be an inevitable and intractable process (for discussion see (Kirkwood and Holliday, 1979, Kirkwood, 2005, Kirkwood, 2008, Munch et al., 2008)), we now know that ageing can be modulated through various environmental, genetic and pharmacological interventions such as dietary restriction (DR) (Kenyon, 2005, Mair and Dillin, 2008, Rikke et al., 2010, Swindell, 2012, Gems and Partridge, 2013). DR is defined as the reduction in overall energy intake, or in specific components of the diet, relative to that consumed normally by individuals with free *ad libitum* (AL) access to food. DR has been conclusively shown to extend lifespan in a wide range of taxonomically diverse organisms (Mair and Dillin, 2008, Selman, 2014). In addition, studies have demonstrated that particular genetic pathways modulate ageing through the observation that deletion of single key genes can extend lifespan and healthspan in model organisms. In particular, reduced signalling *via* the insulin/insulin like growth factor-1 (IGF1) signalling (IIS) pathway or the target of rapamycin (TOR) pathway shows highly conserved effects on lifespan across wide evolutionary distances (Kenyon, 2005, Piper et al., 2008, Broughton and Partridge, 2009, Kenyon, 2010, Selman and Withers, 2011). Indeed, differential expression of several IIS and TOR genes is associated with longevity in humans (Deelen et al., 2013, Passtoors et al., 2013). More recently pharmacological interventions, including rapamycin and metformin, have been shown to extend lifespan in model organisms. This has led to renewed hope in identifying realistic, efficacious and safe pharmacological interventions that will increase the period of our life free from age-related diseases (Selman, 2014). Excitingly, evidence exists that some molecular processes affected by these different interventions may overlap, thereby suggesting some level of commonality (Selman et al., 2009, Yan et al., 2012), although other studies suggest that specific interventions may function through distinct mechanisms (Masternak et al., 2004, Bhattacharya et al., 2012, Fok et al., 2014). Furthermore, recent research has suggested that the effectiveness of each intervention on ageing is highly sensitive to the effects of genetic background. Therefore, the use of such interventions in genetically diverse human beings becomes questionable unless we fully understand the precise role that genetic background plays. In this review, we will discuss the evidence that genetic background plays a role in lifespan extension in response to dietary, genetic and pharmacological interventions in mice.

## Genetic background and dietary restriction (DR)

The first studies on lifespan extension in animals in response to DR were published almost 100 years ago (Osborne et al., 1917, McCay et al., 1935). Consequently, DR is the most reproducible and widely used intervention to modulate lifespan (Masoro, 1995, Speakman and Mitchell, 2011, Selman, 2014). In addition, DR has been shown to induce significant health benefits, with mouse studies showing DR protects animals against a range of age-related and non-age-related pathologies, including various cancers, glaucoma, glucose intolerance and sarcopaenia (Weindruch and Walford, 1982, Sheldon et al., 1995a, Sheldon et al., 1995b, McKiernan et al., 2004, Hempenstall et al., 2010). Furthermore, DR decreases pathogenesis and increases survival in a range of mouse models of disease including Parkinson's disease (Duan and Mattson, 1999), Alzheimer's disease (Halagappa et al., 2007), viral myocarditis (Kanda et al., 2007) and pancreatic cancer (Lanza-Jacoby et al., 2013). The positive effects of DR on health also extend to Rhesus monkeys, where it has been shown that the incidence of cancer, cardiovascular disease, type-II diabetes mellitus and brain atrophy with advancing age are reduced relative to AL controls (Colman et al., 2009, Colman et al., 2014, Mattison et al., 2012). Similarly, DR has been shown to induce significant beneficial effects on metabolism (Weiss et al., 2006) and cardiac function (Stein et al., 2012) in humans, and lowers several risk factors associated with coronary heart disease (Fontana et al., 2007). Despite the large number of studies that have used DR to extend lifespan we still do not understand the precise mechanism(s) through which DR acts to modulate lifespan. In addition, studies using an increasing number of different model systems have implicated that the effects of DR on lifespan extension are not universal and question whether DR is indeed a public modulator of longevity.

Several studies in organisms ranging from yeast to non-human primates have reported no effect of DR on lifespan (Harrison and Archer, 1987, Kirk, 2001, Carey et al., 2002, Forster et al., 2003, Liao et al., 2010, Rikke et al., 2010, Mattison et al., 2012, Schleit et al., 2013). Another caveat has recently emerged from research on mice which indicates that specific genotypes can affect the extent and direction of the lifespan response to DR. As an example, the effect of DR on lifespan in DBA/2 mice has ranged from lifespan extension to lifespan shortening (Fernandes et al., 1976, Turturro et al., 1999, Forster et al., 2003). Clear differences in key variables including terms of husbandry conditions, extent of restriction, diet, gender and age of DR initiation will undoubtedly vary across different studies which may help explain the differences in the findings reported. However, it is also well established that DBA/2 mice show distinct differences in a range of metabolic parameters (e.g., insulin sensitivity, glucose tolerance, metabolic rate) under both AL and DR feeding when compared to strains such as C57BL/6 mice (Funkat et al., 2004, Goren et al., 2004, Berglund et al., 2008, Hempenstall et al., 2010), which may underlie their overall responsiveness to DR. It is also clear that extended lifespan, when observed, in DBA/2 mice in response to DR is more moderate than the extension reported in C57BL/6 mice (Turturro et al., 1999). Recently, lifespan was assayed in heterogeneous ILSXISS recombinant inbred (RI) mice derived from eight distinct mouse strains (Liao et al., 2010, Rikke et al., 2010). In two separate studies undertaken by the Universities of Texas and Colorado, clear lifespan differences existed between distinct ILSXISS lines under 40% DR. The earlier study examined 39 female lines and 41 male lines under DR and reported that only 21% of female and 5% of male lines showed a significant lifespan extension under DR (Liao et al., 2010). In this study, a higher number of lines (26% and 27% for males and females, respectively) showed a significant shortening of lifespan under DR. Similarly, the second study examined 42 female lines again and reported a significant difference in terms of the response to DR across different lines, with 21% of females showing lifespan extension and 19% showing significant truncation of lifespan under DR (Rikke et al., 2010). Similarly, the differential effect of DR on lifespan reported in Rhesus monkeys by the Wisconsin National Primate Centre (Colman et al., 2009, Colman et al., 2014) and the National Institute of Aging (Mattison et al., 2012) may be partly explained by inter-study differences in geographical origin and genetic background of the experimental animals (Mattison et al., 2012; Partridge, 2012; Colman et al., 2014; Selman, 2014). Currently, we do not understand how genetic background may impact on how DR acts to extend life. In ILSXISS mice at least it may be that the optimal DR regime differs between lines and consequently the 40% DR regime employed (Liao et al., 2010, Rikke et al., 2010) was too extreme to maximise lifespan in all lines (Swindell, 2012, Selman, 2014). Furthermore, it is not known if specific lines are more prone to particular pathologies under both AL and DR conditions. However, comparative-type approaches by taking advantage of the differential responses to DR across different mouse strains or RI lines may be a powerful approach to understand precisely how DR acts to slow ageing (Selman, 2014). Consequently, a larger subset of more genetically diverse rodent models should be studied under DR, along with the greater use of non-model organisms to increase our understanding of how DR acts.

## Genetic background and mutant mice

Significant research efforts over the last couple of decades, initially using invertebrate model organisms, have demonstrated that decreased IIS and TOR signalling extends lifespan in a highly conserved fashion across model organisms (Kenyon, 2005, Piper et al., 2008, Gems and Partridge, 2013). In addition, in mice a large number of studies have also shown that specific disruptions in somatotrophic function also extends lifespan significantly relative to wild type controls (Bartke, 2011). Furthermore, it is evident that long-lived genetically mutant mice tend to display a much greater proportion of their life free from age-related pathologies (Selman and Withers, 2011).

As genetic interventions modulating lifespan is a more recent finding compared to studies on DR, it is unsurprising that few studies, so far, have investigated how genetic background can impact on the life-extending effects of specific mutations. The most widely used genetically modified mice in ageing research are those which harbour mutations that result in growth hormone (GH) deficiency or GH resistance (for review see Bartke, 2011, Bartke et al., 2013); Ames mice (Prop1^df^), Snell mice (Pit1^dw^) including growth hormone receptor knockout (GHRKO) and the ‘little’ mouse (GHRHR^lit^). In addition to altered somatotrophic function, these animals appear to have secondary suppression of IIS (Bartke, 2011, Bartke et al., 2013). These models show reproducible lifespan extension across different studies and appear to be protected against a range of age-related pathologies (Brown-Borg et al., 1996, Flurkey et al., 2001, Kinney et al., 2001, Flurkey et al., 2002, Coschigano et al., 2003, Ikeno et al., 2003). The impact of genetic background on lifespan has been studied in Snell mice, where it has been shown that the lifespan extension in these animals is consistent across different genetic backgrounds (Flurkey et al., 2001, Flurkey et al., 2002). However, it should be noted that the additive effects on lifespan with DR reported on a mixed genetic background (Bartke et al., 2001) were lost with a greater penetrance of the C57BL/6J background (Garcia et al., 2008). In addition, Ames mice backcrossed on to a C57BL/6 or 129S1/SvlmJ background led to a significant increase in perinatal mortality (Nasonkin et al., 2004). In 2003, two separate studies were the first to demonstrate that reduced IIS extended lifespan was also conserved in mammals. These studies reported that the loss of the insulin receptor specifically in white adipose tissue (Bluher et al., 2003) or haploinsufficiency in the gene encoding the insulin like growth factor 1-receptor (*Igf1r*^*−/+*^) (Holzenberger et al., 2003) were sufficient to increase lifespan in mice. Holzenberger et al. (2003) showed that female *Igf1r*^*−/+*^ mice maintained on the 129Sv background were 33% longer lived than wild type mice, although the effect in male *Igf1r*^*−/+*^ mice was non-significant (16%). In addition, they showed that female but not male mice were resistant to the effects of the oxidant stressor paraquat. This study has since been criticised due to the short lifespan of the control animals and the small sample size used (Liang et al., 2003). More recently, a second study was undertaken to examine lifespan in *Igf1r*^*−/+*^ mice. Lifespan and end-of-life pathology were examined under housing conditions optimised to maximise the lifespan of the control mice (Bokov et al., 2011). In addition, the *Igf1r*^*−/+*^ mice were re-derived on the C57BL/6 background for at least 10 generations before lifespan was assayed. This second study reported a more modest increase in female lifespan (∼5%; significant using the log-rank test) relative to controls and a slight, but non-significant, reduction in male lifespan. While genetic background and the targeting event used may help explain some of the differences reported between the two studies, the authors in the second study (Bokov et al., 2011) tend to discount this as significant overlap in terms of the metabolic and oxidative stress resistant phenotype which was seen between each study. Interestingly, Bokov et al. (2011) also examined lifespan in *Igf1r*^*−/+*^ female mice on a F_1_ hybrid C57BL/6 × 129Sv background and showed no effect of haploinsufficiency on lifespan. Consequently, the authors of the second study (Bokov et al., 2011) proposed that sub-optimal housing conditions may have led to increased stress exposure of all mice in the earlier study (Holzenberger et al., 2003). This was hypothesized to lead to a survival advantage of the stress resistant *Igf1r*^*−/+*^ females over wild type females, which was not enjoyed by *Igf1r*^*−/+*^ males.

## Genetic background and pharmacological interventions

While evidence suggests DR may not have the ubiquitous effect on lifespan as originally proposed, animals under DR tend to retain a longer period of life free from ill health, including the onset and impact of age-related pathologies such as type-2 diabetes, cardiovascular disease and cancer. Consequently, much current research aims to identify drugs which mimic the effects of DR, i.e., extend vitality in old age. Several compounds have been identified, including rapamycin, metformin and resveratrol, which appear to have significant potential in the development of efficacious and safe DR mimetics (Selman, 2014). However, the precise mechanism through which these interventions act is still unclear and research from murine studies has demonstrated that genotype appears to be important in how particular DR mimetics impact on an individual's phenotype.

**Fig. 1:**
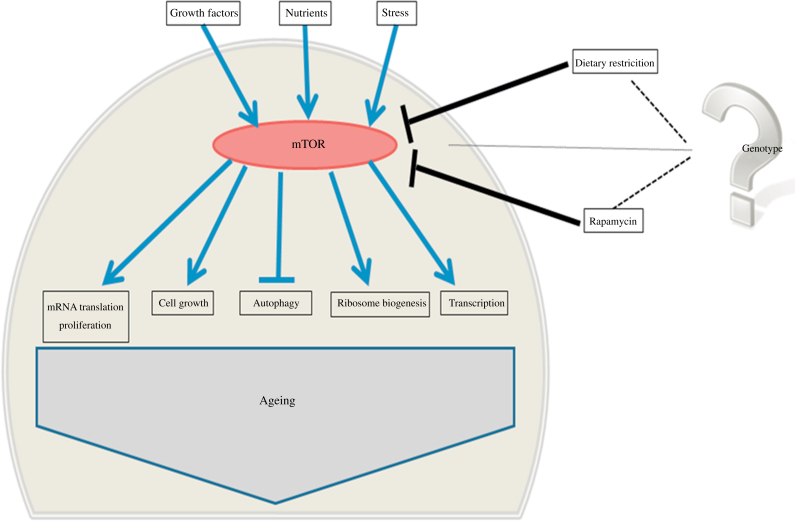
Overview of mammalian target of rapamycin (mTOR) regulated processes.

The TOR pathway appears to be a highly conserved lifespan determinant (Kapahi et al., 2004, Selman et al., 2009, Kapahi et al., 2010, Lamming et al., 2012, Wu et al., 2013), which plays a key role in growth and metabolism (Bjedov and Partridge, 2011) by responding to a range of stimuli including various growth factors, nutrients and energy status (Fig. 1). TOR kinase, the central component of the TOR pathway, forms two functionally different complexes: TOR complex 1 (TORC1), which plays a central role in regulating cellular processes associated with growth and differentiation, and TORC2 which has a regulatory role in the insulin signalling cascade (Lamming and Sabatini, 2011, Lamming et al., 2013a, Lamming et al., 2012, Selman and Partridge, 2012). Rapamycin, a macrolide compound, has been shown to be a highly potent inhibitor of mTORC1, and more recently also of mTORC2 (Lamming and Sabatini, 2011, Lamming et al., 2012, Selman and Partridge, 2012, Lamming et al., 2013a, Lamming et al., 2013b). In model organisms, rapamycin treatment has been shown to extend lifespan (Harrison et al., 2009, Anisimov et al., 2010b, Bjedov et al., 2010, Anisimov et al., 2011b, Miller et al., 2011, Neff et al., 2013) and in mice can attenuate some, but not all, ageing-related phenotypes (Wilkinson et al., 2012, Neff et al., 2013). However, mouse studies using rapamycin to modulate lifespan have shown that its effects on metabolism appear to be highly sensitive to genetic background. As an example, rapamycin treatment induces overt insulin resistance in C57BL/6 mice (Lamming et al., 2012) but insulin resistance was not reported in young or old HET3 mice (Lamming et al., 2013a). In addition, rapamycin appears to have temporal effects on metabolism in mice, leading to hyperinsulinaemia, insulin resistance and glucose tolerance after 2 weeks of rapamycin treatment but hypoinsulinaemia and insulin sensitivity after 20 weeks of treatment (Fang et al., 2013). Recent findings have shown that rapamycin induces sex-dependent effects on lifespan (Miller et al., 2014) and that there appears to be less overlap with DR, in terms of metabolism and transcription, than was originally believed (Fok et al., 2014, Miller et al., 2014). Similarly, the effect of the biguanide metformin on lifespan and health in mice appears to be, at least partly, dependent on genotype, but also dependent on sex and age at which metformin treatment is initiated (Anisimov et al., 2005, Anisimov et al., 2008, Anisimov et al., 2010a, Anisimov et al., 2011a).

## Concluding remarks

It is clear that the dramatic rise in the proportion of elderly individuals making up our population is going to have significant ramifications. Ageing is associated with a decrease in the quality of life linked to an increase in the risk of developing a range of pathologies, including various dementias, type-2 diabetes, osteoporosis, many cancers and cardiovascular disease. Consequently understanding the fundamental processes that drive ageing and increase the susceptibility to develop disease is undoubtedly one of the greatest current challenges in biomedical research. To add to this challenge, it is becoming increasingly prevalent in the literature that genetic heterogeneity is likely to play a critical role in the response to interventions which modulate lifespan and healthspan. Another potential confounding issue is the focussed use of model organisms in such studies, for example, the small number of mouse strains used in ageing research has been highlighted as a potential limitation to the identification of longevity-associated genes (Yuan et al., 2013). Future research efforts should perhaps complement model organism studies with those examining ageing in non-model organisms (Selman et al., 2012), for example, employing comparative-type approaches to study ageing in fast-ageing and slow-ageing species (Austad, 2010a, Austad, 2010b). Additionally, we should also take greater advantage of the inherent differences in longevity and pathology at death seen across different mouse strains (Storer, 1966) to further understand how specific interventions, including DR, genetic inactivation of IIS/mTOR and rapamycin treatment, act to modulate lifespan and health in the background of increasing genetic heterogeneity.
